# Interventions to Promote Civic Engagement Among Youth and Its Outcomes on Mental Health: A Scoping Review

**DOI:** 10.3390/children12060665

**Published:** 2025-05-22

**Authors:** Marina Oubiña López, Diego Gómez Baya

**Affiliations:** 1 Department of Psychology, Universidad Loyola, 41704 Sevilla, Spain; moubinalopez@al.uloyola.es; 2 Department of Social, Developmental and Educational Psychology, Universidad de Huelva, 21004 Huelva, Spain

**Keywords:** civic engagement, well-being, adolescents, program, scoping review

## Abstract

**Background/Objectives**: Youth mental health significantly impacts future well-being, with nearly half of mental health disorders emerging during adolescence. Civic engagement is defined as active participation in the community to improve conditions for others or to help shape the community’s future. It offers a unique opportunity to enhance youth mental well-being, acting as a protective factor against mental health struggles. In this line, Relational Developmental Systems Theory posits that positive youth development is positively linked to civic engagement. The main objective of this literature-based paper is to identify, select, assess, and synthesize the existing literature on interventions promoting mental health in the youth through civic engagement, resulting in an up-to-date review. **Methods**: Following PRISMA guidelines, a search was conducted using PsycInfo, Scopus, and Web of Science to gather studies published between 2018 and 2023, based on the combination of key terms: (“civic engagement” OR “social participation”) AND (“mental health” OR “psychological adjustment” OR “well-being”) AND (adolescen* OR teen* OR youth OR “young people”) AND (program* OR intervention OR training OR education). Data extraction and risk-of-bias assessments were performed. **Results**: Ten studies were included in this review which suggest that civic engagement programs improve youth mental health outcomes, including reduced anxiety, reduced sadness, and increased resilience. These programs foster empowerment, sense of belonging, and social connections, while also enhancing interpersonal skills and career aspirations. Youth also gain valuable skills such as leadership, communication, and problem-solving, contributing to educational and vocational growth. However, challenges such as socio-economic barriers and attendance issues can impact some outcomes, with variations in effectiveness across programs. **Conclusions**: Youth civic engagement programs should integrate mental health support to mitigate the emotional costs of activism, ensuring greater participation and well-being. It is important to adapt these programs to local contexts and provide flexibility to strengthen participation and community impact. Future research should explore the role of cultural, gender, and socio-economic factors in shaping program outcomes and utilize randomized controlled trials to improve the validity and generalizability of findings.

## 1. Introduction

### 1.1. Definitions

Adolescence is a time of rapid biological development and hormonal shifts, referred to as puberty. This phase brings major physical transformations in a teens’ body, as well as social and emotional challenges. There are often a range of difficulties for young people as they navigate these changes [1]. The United Nations (UN) considers adolescence to span from ages 10 to 19. On the other hand, the World Health Organization (WHO) proposes that adolescence should be viewed as occurring between the ages of 10 and 20, acknowledging that, while it begins with puberty, the exact end of this phase is less clear [2]. Young or emerging adulthood is understood to range from about age 18 to 25. However, some researchers contend that this phase can extend until around age 29 [3].

During the adolescence stage, young individuals experience growth in cognitive skills like self-referential processing, decision making, and executive control, promoting their understanding of others’ thoughts and emotions and helping them navigate and interpret the perspectives of those around them more effectively [4].

A youth’s mental health plays a crucial role in their overall well-being, impacting not only during adolescence but also their future mental health into adulthood. In fact, about half of all mental health disorders begin during adolescence [5]. Epidemiological data highlight the significance of this period, indicating that 34.6% of individuals experience the onset of mental disorders before the age of 14. This proportion increases to 48.8% by the age of 18 and further rises to 62.5% by the age of 25 [6]. These statistics underscore the importance of addressing mental health during youth, as the foundation for future well-being is often laid in these early years.

In this context, participation in civic life becomes particularly significant. Engaging in civic activities offers the youth opportunities to enhance their cognitive abilities, social and emotional skills, and sense of self. This involvement allows young people to begin shaping their perspectives, values, and behaviors in ways that carry over into their adult lives [7].

The concept of civic engagement has been widely discussed by scholars. Adler & Goggin [8], after reviewing existing definitions, define it as “how an active citizen participates in the life of a community in order to improve conditions for others or to help shape the community’s future”.

Similarly, the American Psychological Association (APA) defines civic engagement as a set of actions, both individual and collective, aimed at identifying and solving problems of public interest [9]. This positive engagement can manifest itself in various ways, including personal volunteering or participation in community organizations. It may involve direct efforts to address issues, collaboration with others in the community to find solutions, or interaction with democratic institutions. Examples of such engagement include joining a neighborhood association or voting in an election.

Despite these variations in definition, both perspectives share a key principle: an engaged citizen must possess the ability, drive, and opportunity to engage in various forms of civic acts, adapting to different contexts to effectively contribute to societal change [9].

### 1.2. Importance of Civic Engagement

The definitions of civic engagement provided by scholars highlight its role in fostering active participation within communities to improve societal conditions. However, beyond its societal benefits, there is increasing evidence that civic engagement also plays a significant role in mental health [10]. It enhances individual development, especially during adolescence and young adulthood [11]. Engaging in such activities can improve youth mental well-being, serving as a protective factor against mental health struggles [12].

Civic engagement helps build stronger relationships and social networks, providing support from adolescents’ communities. These social ties can be crucial for adolescents, offering more emotional support and reinforcing a sense of belonging. These relationships, in turn, contribute to better mental health outcomes, including higher psychological well-being and reduced symptoms of depression [13].

On a psychological level, civic engagement fosters a sense of benevolence, developed through helping, bonding to, and connecting to others. Active involvement can lead to higher empowerment, improvements in self-perception, and stronger mutual support [11]. Moreover, it is positively associated with youths’ sense of life’s meaning, where adolescents who engage in volunteering report a stronger sense of meaning. At this regard, perceived support may play a critical role in promoting a sense of meaningfulness, further enhancing the mental well-being of those involved [14].

### 1.3. Impact on Positive Mental Health

Positive Youth Development (PYD) offers a framework that aligns well with the benefits of civic engagement. PYD emphasizes the adolescents’ strengths that increase their social engagement and foster the development of healthy behaviors [15]. In contrast to traditional deficit-based models [16,17], PYD encourages youth to take an active role in seeking and utilizing resources that promote their individual skills, strengths, interests, and potentials [18,19].

The “Five C’s” model of PYD [20] identifies five interconnected elements that foster positive youth outcomes: competence, confidence, character, connection, and caring. Competence refers to the ability to effectively navigate various aspects of life, while confidence involves a sense of self-worth and efficacy, and the belief in one’s ability to make meaningful contributions. Connection emphasizes the importance of a sense of belonging and forming positive relationships with others. Character involves adhering to societal and cultural norms, having a strong moral foundation, and taking responsibility for one’s actions. Caring highlights empathy, sympathy, and a commitment to social justice. When these five components are developed, a sixth C emerges (contribution), which is related to contributing to the self, community, and society.

To better understand the dynamics behind PYD, the Relational Developmental Systems Theory (RDS), an extension of the Developmental Systems Theory (DST), offers a valuable framework to describe both the antecedents and consequents of PYD. RDS integrates various factors influencing youth development by explaining how behaviors are interrelated, the factors that drive behavioral change, and the approaches necessary for studying human development. This metatheory lies in the notion that development is the result of the interaction between individuals and their environments. Thus, human behavior cannot be appreciated in isolation, it can only be understood through the relationships established between individuals and their contexts [21]. RDS describes the internal and external assets required to experience PYD and integrate the thriving outcomes of PYD.

**Figure 1:**
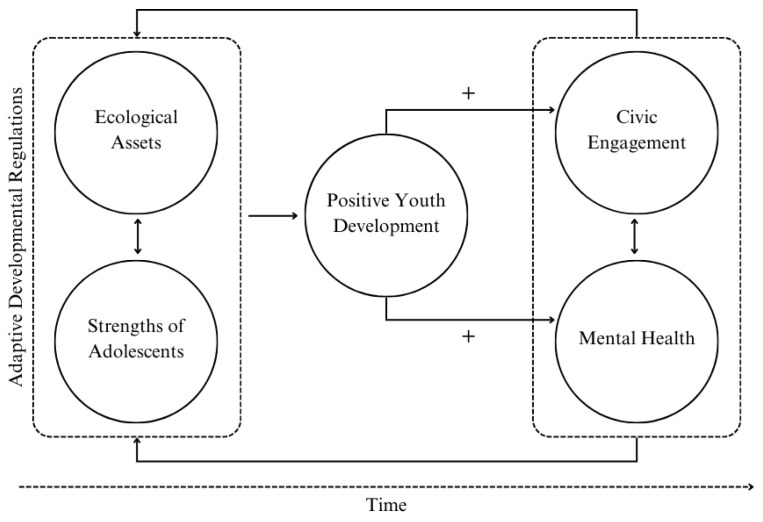
An RDS-based model of the development of civic engagement (adapted from [22]).

Specifically, the RDS-based model proposed by Lerner et al. [22] has been adapted to theoretically frame the development of civic engagement under the PYD model. As seen in Figure 1, the developmental process considers adaptive developmental regulations between ecological assets and youths’ strengths. These adaptive–interactive relationships between the individual and the context are expected to be mutually beneficial and nurture the emergence of the Five C’s of PYD, which in turn contribute to positive outcomes, such as increased civic engagement and mental health development.

### 1.4. The Present Study

For the present study, previous research exploring interventions that support mental health and social participation in adolescents and youth has been taken into consideration. Cahill et al. [23] published a systematic review of studies up to 2017, focusing on activity- and occupation-based interventions that support mental health, positive behavior, and social participation in children and youth. While this study shares similarities with the current study, it primarily focused on interventions rooted in occupational therapy. In contrast, this paper will examine a broader range of approaches, aiming to promote positive development in youth beyond the scope of occupational therapy-based interventions.

The main objective of the present paper is to identify, select, evaluate, and synthesize the most recent literature on interventions aimed at promoting mental health in teenagers and the youth through civic engagement. This study seeks to explore the effectiveness of such interventions, highlighting their potential in fostering positive mental health outcomes and well-being among the youth. A scoping review was conducted to reach this aim, because it is a form of knowledge synthesis that provides an overview of a broad topic like civic engagement, by identifying and mapping the existing literature using different designs and methods. Also, this technique is often used for complex and emerging topics, that still require the identification of research gaps. This scoping review aimed to examine the literature after 2017, because previous works were already documented by Cahill et al. [23].

## 2. Materials and Methods

This scoping review of the literature was based on the PRISMA quality criteria [24], following the PRISMA Extension for Scoping Reviews (PRISMA-Scr) [25] (see Table A1 in the Appendix A). No research protocol was formulated prior to the conduct of this study. Therefore, this review is not registered.

Three databases were consulted: PsycInfo, Scopus, and Web of Science. The searches were carried out from 14 May 2024 to 14 September 2024, based on the combination of key terms: (“civic engagement” OR “social participation”) AND (“mental health” OR “psychological adjustment” OR “well-being”) AND (adolescen* OR teen* OR youth OR “young people”) AND (program* OR intervention OR training OR education).

The inclusion and exclusion criteria were then defined, as shown in Table 1. Articles published between 2018 and 2023 were analyzed to ensure coverage of all the relevant publications of the year, since the database search has been conducted prior to the end of 2024. Articles published before 2018 were excluded, as they have already been extensively reviewed in similar existing research by Cahill et al. [23]. Therefore, limits were used in the search to filter articles published before 2018. Articles using quantitative, qualitative, or mixed methods were included.

Subsequently, a screening of the identified articles was conducted to determine those eligible for inclusion in the review. Initially, articles considered irrelevant based on their titles were excluded. Following this initial selection, a secondary screening involved an evaluation of the abstracts of the remaining articles. Those that did not align with the predetermined eligibility criteria were further excluded from consideration.

Upon selection of the articles, data extraction was performed using a structured coding template. This template facilitated the systematic collection of various data points from the included studies, encompassing the following elements: sample characteristics, research objectives, method, evaluation, and results. Narrative qualitative synthesis methodology was used to create a broader understanding of the phenomenon of youth civic engagement by connecting individual studies and their outcomes.

A critical appraisal of the included sources was conducted using the Joanna Briggs Institute critical appraisal checklist for critical and interpretive research [26], the Mixed Methods Appraisal Tool [27], the Methodological Index for Non-Randomized Studies [28] and the Cochrane Collaboration’s risk-of-bias tool [29]. The academic relevance was analyzed through Journal Citation Reports.

## 3. Results

**Figure 2:**
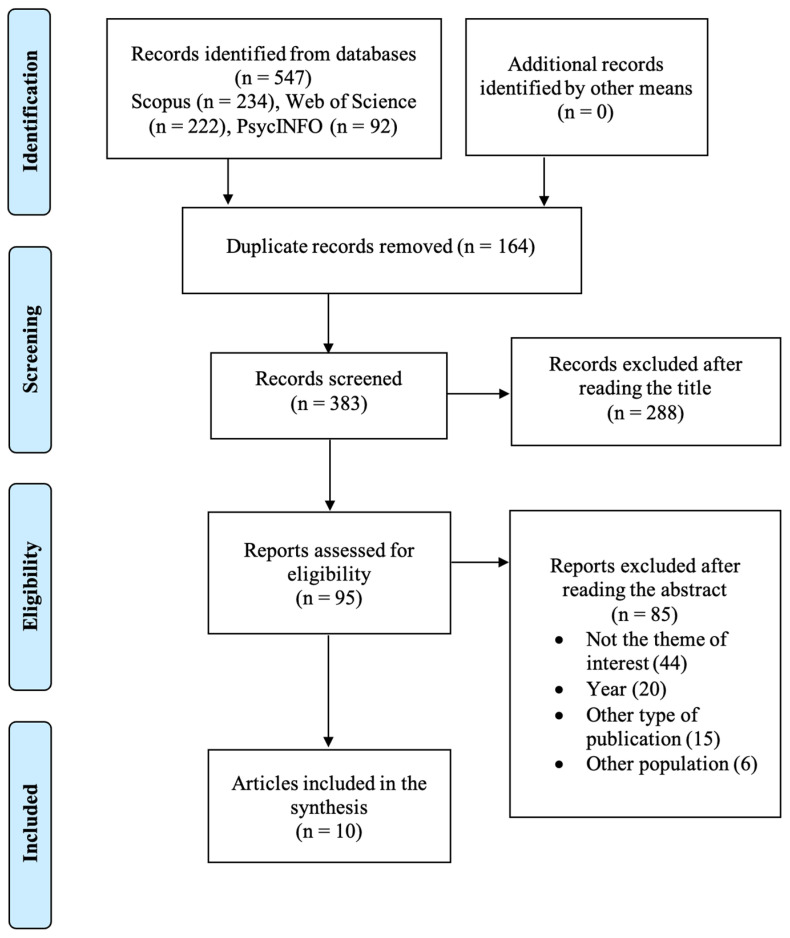
PRISMA flow diagram of the development of the literature selection.

Following a search across multiple databases, a total of 547 records were initially identified. After eliminating 165 duplicates, a total of 383 unique records remained. A preliminary screening based on title assessment led to the exclusion of 288 records. Subsequently, a more detailed abstract review was conducted on the remaining 95 articles assessing its eligibility. At this stage, 85 articles were deemed ineligible, with the reasons for exclusion detailed below. Ultimately, ten studies met the predefined eligibility criteria and were included in this quantitative and qualitative synthesis. A visual representation of this selection process is provided in Figure 2.

### 3.1. Description of Articles

This scoping review synthesizes the findings from ten English-language articles published between 2018 and 2023. The distribution of publications across these years is as follows: two published in 2018, one in 2019, one in 2020, two in 2021, three in 2022, and one in 2023.

Regarding participant demographics, six studies focused on adolescents under the age of 18, while the remaining four included both minors as well as youth up to the age of 25. Geographically, five studies were conducted with participants in the United States and three were based in Europe: one in Italy, one in Portugal, and another one with both Portuguese and Polish youth. There was also one study with participants in Australia, and another one with participants in India. There were five studies who targeted a specific at-risk or marginalized population. Two studies included participants with mental health conditions or disorders, one study focused on marginalized adolescents, another on Asian Americans living in the United States, and another on refugee students.

Table 2 below provides a qualitative and descriptive overview of the articles selected for this review, according to the chronological order of the publications.

### 3.2. Critical Appraisal and Risk of Bias

To assess the appraisal and risk of bias of the qualitative studies included in the review, the Joanna Briggs Institute (JBI) critical appraisal checklist for critical and interpretive research was used [26]. The information gathered is shown in Table 3.

The scoring system for the JBI critical appraisal checklist is as follows: a score of one is assigned for a “yes” answer, while zero is given for all other responses. Drawing from prior systematic reviews, studies that achieved a JBI score above 70% were considered high quality (low risk of bias). Those with scores between 50% and 70% were rated as medium quality, and studies scoring below 50% were classified as low quality [40]. No remarkable risk of bias was identified in the qualitative works included in the scoping review.

For assessing the risk of bias of mixed methods studies included in the review, the Mixed Methods Appraisal Tool (MMAT) [27] was used. In addition to the mixed method items established in the tool, the qualitative and quantitative items were also utilized, following the recommendations by the User Guide [27]. The information gathered is presented in Table 4. No remarkable risk of bias was identified in the mixed method works included in the scoping review.

Regarding quantitative studies, the Methodological Index for Non-Randomized Studies (MINORS) [28] was applied, and the Cochrane Collaboration’s risk-of-bias tool [29] was used for randomized controlled trials (RCTs). The details of these tools are presented in Table 5 and Table 6, respectively.

The MINORS tool does not define specific cutoffs for determining the risk of bias. However, based on existing studies [41], scores below 8 in non-comparative studies are considered poor quality, indicating a high risk of bias. Scores between 9 and 14 are classified as moderate quality, suggesting a moderate risk of bias, while scores between 15 and 16 are considered good quality, corresponding to a low risk of bias. For non-comparative studies, the maximum score is 16. For comparative studies, where the maximum score is 24, the cutoffs are as follows: scores below 14 indicate a high risk of bias, scores between 15 and 22 indicate a moderate risk, and scores between 23 and 24 indicate a low risk of bias. Regarding the work by Prati et al. [33], only a potential bias regarding not applying a double-blind evaluation was identified.

Cochrane’s risk-of-bias tool for RCTs is applied to individual outcomes, allowing for the assessment of risk of bias for each outcome separately. The tool identifies various potential sources of bias, including selection bias, performance bias, detection bias, attrition bias, reporting bias, and other biases [29]. Only a risk of bias concerning participants’ and personnel’s blinding was identified in Gehue et al. [35].

### 3.3. Academic Relevance

To assess the academic relevance of the ten articles included in this review, a selection of bibliometric data has been collected (see Table 7).

Regarding the relevance of the journals in which the studies included in this revision were mainly published, the Journal Citation Reports indicators corresponding to the journals indexed in the Web of Science (WoS) database were used. More precisely, the categories in which the journals were grouped, their impact factor (according to the Journal Citation Reports, JIF), and the quartile in which each journal is located within its category (Q), as well as its percentile, were considered. Concerning journals included in several categories, the highest category was selected in the description.

The number of citations that each document has received in Google Scholar has been added as an indicator to assess the academic relevance of each literature article, in comparison to other journal articles.

### 3.4. Description of Results

**Figure 3:**
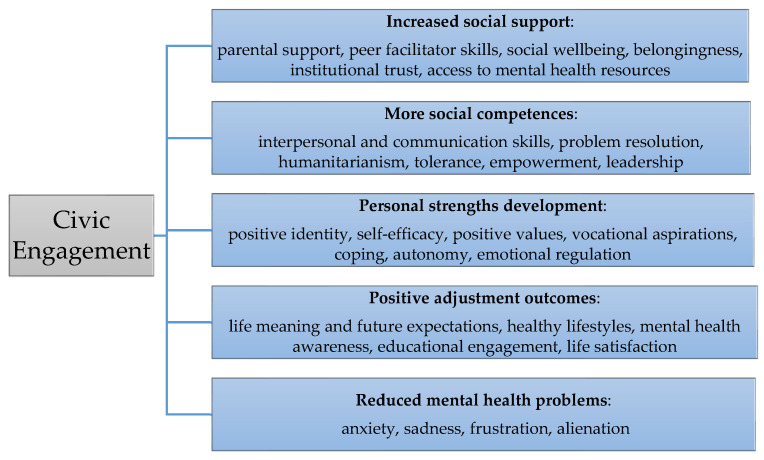
Infographic about the connection between civic engagement activities and some psychological and social benefits.

As Figure 3 shows, the studies highlighted improvements in mental health outcomes [32,39]. Some of these outcomes were reduced anxiety [39] and reduced sadness, due to acquiring emotional management skills, social support, and access to mental health services [32]. Additionally, resilience was shown in navigating issues and overcoming challenges [38]. Some of the challenges or threats identified that could affect youths’ mental health were socio-economic barriers, discrimination, immigrant struggles [38], bullying, lack of acceptance, lack of support, and environmental pressure [33].

One of the most recurring themes from the analyzed articles is empowerment. By participating in civic engagement programs, especially in leadership and community activities, the youth fostered a sense of empowerment. Some of the activities they engaged in were teams, church, and student associations, as well as volunteering, completing solidarity work, and tutoring other people [30].

Most of the participants expressed a sense of pride in the accomplishments they made, accompanied by a strong desire to drive change within their communities [31,36,38]. Some of these changes include addressing substance abuse, littering, and school overcrowding [38].

An increased confidence, a sense of self-efficacy, and an improvement in relationships with peers and other community members were reported across various of the studies analyzed [31,32,39]. Mathias et al. [32] noted in their study specific differences between young men and women in these aspects that foster personal growth. Young men experienced improved perceptions within their communities, while young women gained a greater freedom of movement and improved confidence in communication. In the study carried out by Lin et al. [31], the youth explored their identity by reflecting in the community on their ethnic identity (Asian American) and cultural differences.

The youth expressed different motivations to engage in civic behaviors. Many had a strong sense of duty and empowerment throughout the engagement activities carried out. Some of the activities that fostered these feelings were policy discussions [38], protest involvement, and advocacy training, sometimes driven by a desire to combat xenophobia as well as negative stereotypes [39].

Regarding psychosocial outcomes (see Figure 3), a sense of belonging to the community was a key aspect of youth engagement programs. The youth reported feeling a strong connection to their communities [31] and a sense of pride in contributing to positive community changes [36]. This encouraged engagement in community activities such as volunteering, fostering civic responsibility as well [31].

Concerning social outcomes, the participation in these programs improved interpersonal relationships, as a result of teens improving their communication and social skills, acquiring healthier behavior in regard to social norms, and increasing their social competence [31]. Many of them reported strengthened relationships with family and community members, contributing to an overall improved social well-being. Furthermore, the development of new friendship networks was a common outcome across the studies [32,39]. In addition to these benefits, some participants stated that civic engagement creates a sense of belonging and personal connection [31,39].

The youth gained useful skills for future work and studies, including leadership, competences for problem solving, and communication skills [31]. For instance, skills in advocacy [39], as well as job-related competencies, were developed, contributing to job readiness [31].

Career and educational aspirations were also informed. While some studies found no significant differences in vocational outcomes [35], others noted that the participation and active engagement in community programs led to an increase in career aspirations and outcomes in terms of educational and work aspirations [30,31,38].

Most teens expressed an interest in pursuing education beyond high school [30], with some of them indicating an increased interest in specific careers, like teaching and healthcare, due to the exposure to health careers and mentorship during the programs [31].

Focusing on educational outcomes, the study by Gehue et al. [35] found that there was a notable increase in high school graduation rates, as well as employment rates, especially right after participants’ involvement in youth development programs, since a smaller increase in these rates was seen at follow-up.

Some of the studies identified challenges and contextual factors that made an impact on the outcomes. Among these contextual factors that influenced the outcomes were parental support, peer facilitation skills [32], and socio-economic barriers [38]. An example noted in the investigation by Mathias et al. [32] in India was that strict parental controls were limiting freedom for young women, which affected their participation in some activities, often not being allowed to attend the group since they could not leave their homes without parental permission. Furthermore, family economic status was linked to life satisfaction [33]. In this case, lower-income families reported greater gaps in these satisfaction levels. These disparities in life satisfaction levels showed a bigger improvement in the quality of life between low-economic-status families and average families, than in the transition between average families and those with the highest economic status.

Program barriers were discussed by some facilitators. Challenges were noted related to meeting times and attendance, which impacted the completion of activities. Suggestions for improvement from participants included the offer of a more flexible schedule and longer meeting times to enhance participation and outcomes [37].

With respect to the impact that the programs had and their evaluation, different results were found. In some of the studies analyzed there were no significant changes between pre-test and post-test data in certain measured dimensions. In the study carried out by Branquinho & Gaspar de Matos [30], dimensions such as feelings towards life, humanitarianism, or competencies for problem resolution showed no significant changes. Alegría et al. [36] detected an increase in civic participation and leadership competence when comparing data from the start to the wrap-up of the program; nevertheless, these increases were not maintained at follow-up. Prati et al. [33] found no significant differences between the control and intervention groups in aspects like social well-being and EU engagement mindset. However, they highlighted significant group effects over time, with an improvement in well-being, institutional trust, and political participation noted in the intervention groups.

Activity levels were found to influence the results by Bennett et al. [37]. In this study, the intensity of program activities was a significant predictor of positive outcomes. The programs that were developed in the research paper with higher activity levels resulted in greater success compared with those programs that contained fewer activities and had lower implementation indices. Other aspects that influenced the programs’ effectiveness were group dynamics and participation, peer facilitator support, and gender-transformative approaches [32].

Overall, most programs demonstrated success. Participants felt empowered with a stronger sense of self-efficacy, inter alia, and reduced anxiety levels, although there were variations in programs’ effectiveness.

## 4. Discussion

### 4.1. General Interpretation of Results

The main objective of this paper was to identify, select, evaluate, and synthesize the most recent literature on interventions aimed at promoting mental health in teenagers and the youth through civic engagement. Ten research papers were selected, and the interpretations of their findings are discussed below.

Overall, the findings suggest that youth civic engagement programs have positive effects on mental health [32,38,39] and empowerment [30,31,38]. Alegría et al. [36] noted an increase in psychological distress at wrap-up and follow-up, which, according to participants, did not seem to have been influenced by the civic engagement program, but rather, by school exams and/or family issues occurring at the time of measurement. This made the authors realize that they should focus on well-being, rather than mental health as an outcome.

These programs also seem to have positive social outcomes, where participants experienced the development of a sense of belonging [31,36], improved social relationships [31,32,39] and enhanced leadership, problem-solving, and advocacy skills [31,39]. An increase in career and educational aspirations [31], as well as higher graduation and employment rates [35], have also been reported. However, these effects are not uniform, and certain contextual factors (e.g., socio-economic status and parental support) could influence the extent of these outcomes [32,37,38].

The aspects that were considered effective include: Policy discussions that helped participants understand their local policy issues and explore the context of policy change. The integration of data collection and policy lessons fostered skills like observing, interviewing, and presenting trustworthy data, connecting it to real-world policy advocacy. Group dynamics and interactive activities, such as role-playing, made the learning process engaging and practical, emphasizing empowerment and active participation [32,37]. Small, participatory, and peer-led groups seemed more effective than larger groups, since the latter may limit participation, not achieving as many positive outcomes. Support from peer facilitators and gender-transformative approaches played a key role in the promotion of mental health, social inclusion, and positive behavioral changes. This was particularly impactful for the women in India, who reported an increase in self-efficacy, freedom of movement, and communication [32].

Furthermore, some characteristics were observed to be less effective when delivering the sessions, including meeting time and length. An hour-long session was considered too short to cover all the material, leading to rushed sessions and incomplete learning. Low attendance also had a negative impact on the sessions’ effectiveness, since not everyone was able to benefit from the material equally [37].

### 4.2. Contributions and Practical Implications

One of the implications mentioned in the articles is the role of civic engagement in fostering a sense of empowerment, pride, and belonging. These outcomes seem to be crucial for youth development, as they contribute not only to mental health improvements but also to personal growth and positive community change. Youth-led programs have a positive effect on fostering leadership skills, as well as promoting a sense of civic responsibility [36,39]. These programs provide opportunities for participants to be the agents that promote their communities’ well-being. By doing so, their sense of agency is enhanced, which encourages them to play an active part in the promotion of social change.

Cureton [39] provides evidence that civic engagement enhances critical consciousness, by helping young refugees to reflect on and respond to systemic social injustices, as well as gaining a sense of collective agency. In the critical consciousness framework, the process of critical reflection, critical motivation, and critical action plays an indispensable part in empowering youth to understand their own societal positioning and to act in addressing injustices. These findings suggest that programs that offer the youth autonomy to select their focus areas and engage in meaningful action not only empower them as leaders but also promote a sense of connection to broader societal issues, linking personal growth with societal change.

In a similar way, participatory models such as photovoice, discussed by Koren & Mottola [38], and peer-led programs in Lin et al.’s research [31] emphasize the importance that youth leadership holds in addressing societal issues. These participatory models nurture critical thinking and leadership development, which are central aspects of empowerment and active citizenship. They do so by offering participants a program to document, reflect, and act on issues that are significant to them, promoting their individual well-being and social involvement.

Nevertheless, the relationship established between civic engagement and mental health is not linear, and even though many programs contribute to the flourishing of leadership skills and youths’ social engagement, this empowerment through activism can pose some risks to youths’ emotional well-being. The study carried out by Alegría et al. [36] points out that the emotional cost of activism (additional stressors and responsibilities) can lead to psychological distress, especially when addressing difficult emotional social issues, and if the experience is time consuming, raises questions on how to support emotional resilience through these programs. In a similar way, as mentioned before, in Branquinho & Gaspar de Matos [30], long-term psychological well-being did not improve significantly, which raises concerns regarding the possible emotional costs derived from civic engagement. This suggests that without effective interventions to address the emotional well-being of youth participants, such as relaxation and self-care approaches [36], the risks of disengagement and burnout increase. Thus, programs aimed at promoting youth engagement should also address competence development that enables the youth to deal with emotional distress and caring fatigue, as well as to display the most adaptive coping responses.

Gehue et al. [35] and Gaspar de Matos et al. [34] also highlight the importance of addressing youths’ mental health challenges in the interventions. Both papers emphasize the need to not only offer the youth social and vocational opportunities in the programs, but also a constant support to handle mental health symptoms. As Gehue et al. [35] underline in their study with volunteers that had emerging mental health disorders, symptom remission is important for participants to have a functional recovery, suggesting that poor mental health and the factors associated with it are likely to have residual effects on psychological distress and functioning. This implies that even though the positive developmental impacts of civic engagement are significant, they should be balanced carefully with emotional and mental health support. There is a need for program structures that not only focus on promoting civic engagement but also on integrating other strategies that support youth mental health. Some suggested examples are offering mentorship, self-care workshops, and access to counseling services. In this way, burnout can be prevented, ensuring that the youth are able to sustain their activism without adverse consequences. These findings underscore the importance of the integration of mental health resources in civic engagement programs to address the emotional challenges that the youth face.

Some authors describe that one key element in the success of these civic engagement programs is the role of adult facilitators. Both Bennett et al. [37] and Alegría et al. [36] emphasize how adult guidance helps the youth navigate the complexities of social advocacy and leadership. These studies underscore the need for supportive adults who can offer emotional and practical advice, especially in challenging circumstances. As seen in the investigations, youth–adult collaboration contributes to strengthening the continuity of youth participation. It also fosters a supportive environment where young people feel empowered to continue their advocacy work, making them feel valued and capable of making an impact on their communities in the long term.

Another central theme across the studies is the need for flexibility in youth civic engagement programs. The EYPC program by Bennett et al. [37] and the youth-led initiatives by Alegría et al. [36] demonstrate the benefits of adjusting programs to local needs and community contexts. In the program by Bennett et al. [37], a range of issues were addressed, such as tobacco control or alcohol regulations. This ensured the youth engaged with causes that directly affected their lives. Similarly, the initiative by Alegría et al. [36] allowed the participants to select their own focus areas. This strengthened the youths’ sense of ownership and investment in what they were doing. This adaptability is particularly relevant in culturally diverse settings, as seen in the investigation by Mathias et al. [32]. In their research, it was found that gender-sensitive and culturally tailored interventions were especially effective in promoting social inclusion and mental welfare amongst marginalized groups.

These results imply that the adaptability or flexibility of the programs to address specific community contexts plays a part in helping the youth to connect more with the issues they are addressing, which reflect their lived experiences, resulting in a more meaningful and sustained engagement. The combination of mental health support with civic engagement may also increase the effectiveness of the interventions, by integrating personal skills development with nurturing contexts that foster youth participation and empowerment. This synergetic association between social engagement and mental health is consistent with RDS theory. The framework integrates the adaptive regulations between the youth and their context in a virtuous cycle characterized by the interaction between PYD and social contribution.

### 4.3. Limitations of the Studies Included in the Review

Based on the findings from these studies, several recommendations emerge for future practice and research.

There is a need for longitudinal studies to better understand the long-term impacts of youth civic engagement on both individual well-being and community change [32,33,36]. Some programs report short-term successes, but they lack data, or their data lead to inconsistent results [30] on the sustainability of these impacts.

Research works with bigger samples are needed to build evidence, since applying findings to the larger population is difficult with small samples [30,31,34,38,39]. Small sample size has also limited the ability to determine significant associations between the program experiences of the youth and development outcomes [31]. Sample diversity might have also impacted the findings’ consistency, since participants were diverse in age, sex, problems, culture, and backgrounds, and took part in different program experiences [31,32,33]. Cultural and demographic factors such as ethnicity were not taken into account, which limited the research regarding the influence that these factors could have on social functioning [35].

There is a strong need for program adaptability, particularly in diverse cultural contexts, so that the youth are engaged in ways that resonate with their lived experiences and community needs. Research should include specific cultural, gender, and at-risk populations, as current studies show that demographic factors like ethnicity, socio-economic status, and gender are able to significantly influence program outcomes. To improve the applicability and relevance of these programs, future investigations should explore how these variables interact with program experiences and outcomes. Furthermore, future studies should integrate culturally sensitive approaches that address the unique needs and challenges faced by marginalized and vulnerable youth. This procedure will ensure that civic engagement programs are both contextually appropriate and effective, fostering positive developmental outcomes across diverse populations.

Studies suggest the presence of sampling bias, since methods such as snowball sampling might lead to a non-representative sample with a self-selection tendency, limiting the generalization of results [31,35,37]. Randomization procedures were not viable for the studies’ designs, risking the presence of selection bias [34].

Many programs used individual interviews or self-reporting questionnaires as methods for data collection. This was subject to response biases such as social desirability, exaggerating positive outcomes and under-reporting negative experiences, as well as a recall tendency, especially when the recall happened further away in time from the program or if the participant was not as engaged [30,31,32,33].

It was noted that some programs did not include a control group [35]. Only one randomized controlled trial was included in the review, what may limit the generalization of this study’s outcomes. Therefore, it is not feasible to separate the interventions’ effects from the standard or usual (control) treatment. The use of randomized controlled trials is recommended in order to increase the validity of the conclusions.

### 4.4. Limitations of the Scoping Review and Future Research

In accordance with what has been researched, it is important to point out the limitations found during the present scoping review, as well as its future lines of investigation.

The first limitation of this paper is that only reports in English were included in the review, which limits the breadth of information that can be assessed. Articles published in other languages could offer valuable insights, allowing for a richer comparison of how civic engagement is approached in different cultural contexts and countries. Also, potential publication bias should be acknowledged from the limited number of selected studies, which may reduce the impact of our conclusions.

A second limitation is that only three databases were consulted during the search process. While these databases provided a substantial number of relevant studies, expanding the search to include additional databases could have yielded a greater number of articles. This might have helped to identify studies that were not indexed in the selected databases, further enriching the findings and broadening the evidence base.

A methodological limitation of this review is the absence of a pre-registered protocol, which would have reduced bias and enhanced transparency. However, due to time constraints, protocol registration was not feasible. This omission may affect the reproducibility and perceived rigor of the review.

Finally, while narrative synthesis is a suitable and widely accepted approach for scoping reviews, it is inherently more interpretive than quantitative synthesis methods. As such, the integration of the findings may be influenced by subjective interpretation and statistical comparisons (such as effect sizes) cannot be made across the studies. Thus, the methodological differences among the studies included in this review may make it difficult for the integration of the results and the strengths of our conclusions.

## 5. Conclusions

The reviewed studies highlight the significant positive effects of civic engagement programs on youth mental health, empowerment, and personal growth. These programs not only improve mental welfare outcomes, such as reduced anxiety and sadness, but also foster resilience, confidence, and a sense of pride through active participation in leadership and community activities. However, the effectiveness of these programs is influenced by contextual factors, including socio-economic barriers and parental support, emphasizing the need for adaptable and culturally sensitive approaches. Lastly, while youth civic engagement has substantial benefits, it is crucial to integrate mental health support within these programs to prevent burnout and ensure sustained positive outcomes. By striking a balance between empowerment and emotional well-being, these programs can better support youth in their personal and community-driven endeavors.

## Figures and Tables

**Table 1:** Inclusion and exclusion criteria.

	Inclusion Criteria	Exclusion Criteria
Population	Adolescents and young adults (age 10 to 25)	Minors younger than 10 and adults older than 25
Type of publication	Original articles involved in a peer-review process Available and/or open-access articles Use of an intervention	Others such as: Editorials or expert opinions Systematic or narrative reviews Dissertation, theses, presentations, and conference proceedings Non–peer reviewed publications Unavailable or paid articles
Study design	Quantitative, qualitative, or mixed	Others such as: case studies
Language	English	Other
Publication date	2018 to 2023	Earlier than 2018 Later than 2024
Results	Relationship between the programs carried out to promote civic engagement and its outcomes on the participants’ mental health	Other

**Table 2:** Description of the articles included in this scoping review.

Study ID	Sample	Method	Main Results
Branquinho & Gaspar de Matos (2018) [30]	*n* = 46 Youth participants aged 11 to 18 (M = 16.13; SD = 1.89) Portugal	Longitudinal qualitative research	(1) Five dimensions were obtained: feelings and competencies for action, interpersonal skills, competencies for problem resolution, humanitarianism, and feelings towards life. (2) Teens considered that their participation was a valuable significant asset. (3) Pre-post data evaluation showed no significant differences across the five dimensions from year 1 to year 2. (4) Most participants engaged in different community leadership activities and volunteering. (5) Future plans: the majority intended to continue education beyond high school, with an increased interest in jobs in year 2.
Lin et al. (2018) [31]	*n* = 18 Asian American participants aged 18 to 33 at the time of interview but aged 13 to 24 (M = 16) at initial involvement United States	Qualitative research analyzing in-depth interviews	Key components of positive youth development programs: (1) Opportunities for skill building and participation. (2) Positive social norms: healthy behaviors. (3) Safe space: for self-expression and identity exploration. (4) Sense of belonging. (5) Supportive organizational culture and staff. Influence on youth development: (1) Identity development. (2) Healthy life choices. (3) Improved relationships. (4) Competence and self-efficacy. (5) Career choice. (6) Community involvement and volunteerism.
Mathias et al. (2019) [32]	*n* = 142 Youth under 25 years old (M = 18.9) affected by psycho-social disability India	Longitudinal mixed-method research	(1) Formation of new friendship networks. (2) Increased self-efficacy and confidence. (3) Improved mental health: reduced anxiety, sadness, and frustration. (4) Increased community participation. (5) Women increased freedom of movement and confidence in communication. (6) Men improved community perceptions. (7) Contextual and intervention factors influencing outcomes: parental support, peer facilitator skills, limited freedom for young women, and socio-economic factors.
Prati et al. (2020) [33]	*n* = 69 Italian high school students 15 to 17 years old (M = 15.74; *SD* = 0.50) at pretest Italy	Longitudinal quantitative research	(1) No significant differences between the control and intervention groups in social well-being (*p* > 0.05), European identification (*p* > 0.05), attitudes toward the EU (*p* > 0.05), political alienation (*p* > 0.05), institutional trust (*p* > 0.05), and EU-level participation (*p* > 0.05). (2) Significant group × time interactions were observed for political alienation, institutional trust, EU-level participation, and social well-being. These effects had medium to large effect sizes. (3) A median split based on European identification showed no significant differences in post-test measures of the same outcomes between those with higher or lower identification as European.
Gaspar de Matos et al. (2021) [34]	Quantitative Study: *n* = 10,571 Adolescents aged 11 to 15 Qualitative Study: *n* = 72 Adolescents aged 14 to 16 from Portugal and Poland	Longitudinal mixed-method research	(1) Health-related issues increase with age and are more prevalent among boys. (2) Life satisfaction scores were similar, but distribution varied. Family economic status significantly influenced life satisfaction. (3) Polish adolescents reported more psychosomatic complaints than Portuguese. Qualitative results on the view of mental health: (1) Positive feedback about the clarity of the questions. (2) Linked mental health to both positive and negative feelings. (3) Identification of factors for maintaining mental health. (4) Identification of threats. (5) Desire for more attention to specific mental health issues in research and educational programs.
Gehue et al. (2021) [35]	*n* = 133 Youth aged 14 to 25 with emerging mental health disorders Australia	Longitudinal quantitative research	(1) SOFAS (Social and Occupational Functioning scale): scores improved (B = 4.96, *p* < 0.0001), but there were no significant differences by randomization group or diagnosis. (2) FAST (Functional Assessment Short Test): a decrease in scores indicated improved functioning (B = −3.44, *p* = 0.0001), but no significant associations with randomization group or sessions attended were found. (3) BDQ-7 (“Days unable”): scores decreased over time (B = −2.01, *p* < 0.0001), but no significant associations were found. (4) BDQ-8 (“Days in bed”): scores also decreased (B = −1.06, *p* = 0.023), but there were no significant associations with attendance, randomization group, or diagnosis. (5) Educational engagement improved significantly by the trial’s end. However, no significant differences were found in vocational outcomes between the trial’s end and follow-up.
Alegría et al. (2022) [36]	*n* = 19 Youth participants aged 14 to 19 years (M = 16.11; SD = 1.10) United States	Longitudinal mixed method research	(1) Desire for change. (2) Sense of pride. (3) Power and responsibility to enact community changes. Regarding the survey, two outcomes had a significant increase from baseline to wrap-up. These two are civic participation (*p* = 0.033) and leadership competence (*p* = 0.021). However, the effects were not sustained at follow-up. There was also a marginal increase in belief in self at wrap-up (*p* = 0.081); however, it was not statistically significant.
Bennett et al. (2022) [37]	*n* = 455 Youth participants, middle and high school students (M = 16.01; SD = 1.43) Illinois, United States	Longitudinal mixed method research	Quantitatively, there was an overall program success, with an overall attrition rate of approximately 20%. Significant differences were noted in outcomes based on activity levels. Significant correlations were found between outcome scores and the number of activities (r = −0.54, *p* < 0.01) and implementation indices (r = −0.47, *p* < 0.01). The number of activities implemented was a significant predictor, explaining 31% of the variation. Facilitators reported on successes and challenges in five areas: (1) Most effective aspects: discussions on local policies and data collection. (2) Least effective: concerns about meeting times and attendance. (3) Participants’ likes: policy discussions and community engagement. (4) Participants’ dislikes: no major dislikes were reported. (5) Suggestions for improvement: longer meeting times and more flexible scheduling.
Koren & Mottola (2022) [38]	*n* = 11 High school marginalized teens from grade 10 to 12 Massachusetts, United States	Qualitative research using focus group photograph and narrative analysis	(1) Teens gained empowerment and a deeper connection to their community. (2) They explored and reflected on their identities. (3) The diverse group discussions broadened participants’ perspectives. (4) The photovoice activity empowered teens. (5) Teens shared challenges like discrimination, immigrant struggles, and socioeconomic barriers. (6) Participants expressed a desire to drive change in their communities. (7) Teens shared aspirations for academic and career success.
Cureton (2023) [39]	*n* = 15 Refugee students in U.S. high schools aged 14 to 17 Chicago, United States	Qualitative research	(1) Motivations for civic engagement: sense of duty to learn about and advocate for their rights, driven by a desire to combat xenophobia and stereotypes. (2) Sense of duty. (3) Community connection. (4) Civic training programs. (5) Personal empowerment: civic involvement as a coping mechanism in response to anxiety. (6) Building a supportive community.

**Table 3:** JBI critical appraisal checklist for qualitative research [26].

Study ID	1	2	3	4	5	6	7	8	9	10	JBI Score	Risk of Bias
*Qualitative Research*												
Branquinho & Gaspar de Matos (2018) [30]	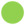	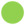	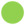	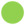	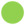	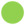	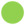	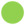	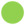	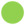	100%	Low
Lin et al. (2018) [31]	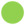	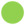	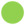	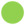	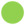	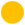	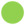	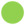	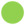	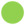	90%	Low
Koren & Mottola (2022) [38]	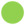	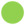	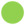	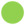	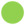	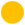	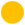	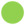	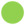	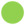	80%	Low
Cureton (2023) [39]	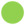	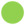	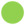	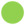	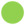	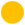	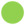	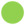	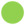	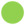	90%	Low
*Mixed Methods Research*												
Mathias et al. (2019) [32]	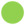	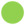	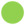	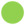	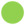	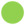	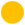	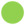	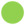	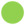	90%	Low
Gaspar de Matos et al. (2021) [34]	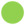	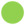	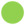	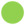	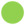	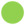	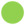	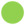	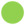	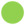	100%	Low
Alegría et al. (2022) [36]	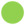	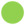	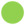	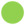	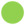	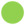	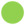	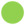	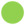	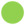	100%	Low
Bennett et al. (2022) [37]	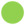	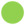	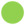	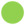	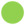	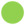	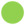	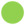	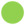	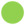	100%	Low

Note: 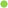 = Yes. 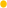 = Unclear. 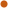 = No. NA = Not Applicable.

**Table 4:** Mixed Methods Appraisal Tool (MMAT) [27].

Study ID	Screening		Qualitative					Quantitative Descriptive					Mixed Methods					MMAT Score	Risk of Bias
	S1	S2	1.1	1.2	1.3	1.4	1.5	4.1	4.2	4.3	4.4	4.5	5.1	5.2	5.3	5.4	5.5		
Mathias et al. (2019) [32]	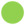	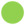	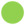	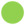	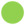	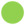	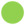	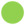	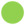	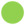	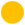	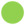	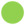	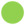	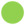	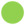	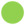	80%	Low
Gaspar de Matos et al. (2021) [34]	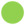	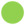	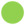	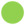	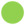	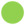	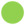	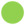	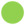	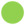	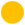	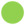	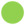	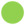	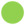	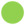	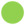	80%	Low
Alegría et al. (2022) [36]	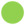	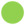	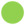	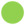	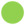	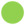	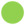	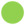	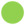	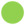	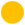	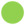	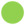	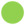	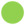	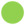	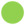	80%	Low
Bennett et al. (2022) [37]	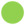	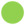	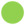	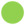	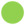	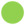	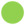	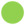	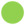	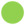	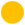	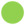	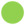	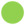	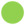	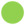	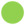	80%	Low

Note: 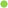 = Yes. 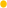 = Cannot tell. 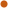 = No.

**Table 5:** Risk of bias non-randomized studies (MINORS) [28].

Study ID	1	2	3	4	5	6	7	8	9	10	11	12	Quality Score	Risk of Bias
Prati et al. (2020) [33]	2	2	2	2	0	2	1	2	2	2	2	2	21/24	Moderate

Note: 0 = Not reported. 1 = Reported but not precise. 2 = Precise.

**Table 6:** Risk of bias randomized controlled trials [29].

Study ID	Sequence Generation (Selection Bias)	Allocation Concealment (Selection Bias)	Blinding of Participants and Personnel (Performance Bias)	Blinding of Outcome Assessment (Detection Bias)	Incomplete Outcome Data (Attrition Bias)	Selective Outcome Reporting (Reporting Bias)	Other Potential Threats to Validity (Other Bias)
Gehue et al. (2021) [35]	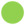	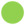	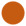	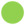	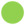	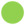	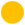

Note: 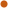 = High risk. 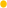 = Uncertain risk. 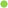 = Low risk.

**Table 7:** Bibliometric data of the review documents.

Study ID	Journal	Category	Journal Impact Factor (JIF)	Quartile (JIF)	Percentile (JIF)	Citations Received (Article)
Branquinho & Gaspar de Matos (2018) [30]	Child Indicators Research	Social Sciences, Interdisciplinary	1.656	Q2	62.98	26
Lin et al. (2018) [31]	Children and Youth Services Review	Social Work	1.684	Q1	84.88	12
Mathias et al. (2019) [32]	Global Public Health	Public, Environmental, and Occupational Health	1.791	Q2	55.85	22
Prati et al. (2020) [33]	Health Education & Behavior	Public, Environmental, and Occupational Health	2.623	Q2	56.53	37
Gaspar de Matos et al. (2021) [34]	Journal of Community Psychology	Psychology, Multidisciplinary	2.297	Q3	46.28	4
Gehue et al. (2021) [35]	Journal of Affective Disorders	Psychiatry	6.533	Q1	80.07	4
Alegría et al. (2022) [36]	American Journal of Community Psychology	Psychology, Multidisciplinary	3.1	Q2	66.3	7
Bennett et al. (2022) [37]	Children and Youth Services Review	Social Work	3.3	Q1	92.9	1
Koren & Mottola (2022) [38]	Journal of Community Psychology	Psychology, Multidisciplinary	2.3	Q3	49.3	4
Cureton (2023) [39]	Children & Schools	Social Work	1.2	Q3	46.2	2

## Data Availability

The original contributions presented in the study are included in the article, further inquiries can be directed to the corresponding author.

## Abbreviations

The following abbreviations are used in this manuscript:

UN	United Nations
WHO	World Health Organization
APA	American Psychological Association
PYD	Positive Youth Development
RDS	Relational Developmental Systems Theory
DST	Developmental Systems Theory
JBI	Joanna Briggs Institute
MMAT	Mixed Methods Appraisal Tool
MINORS	Methodological Index for Non-Randomized Studies
RCT	Randomized Controlled Trials
JIF	Journal Impact Factor

## Appendix A

**Table A1:** Preferred Reporting Items for Systematic reviews and Meta-Analyses extension for Scoping Reviews (PRISMA-ScR) checklist [25].

SECTION	ITEM	PRISMA-ScR CHECKLIST ITEM	REPORTED ON PAGE
TITLE			
Title	1	Identify the report as a scoping review.	1
ABSTRACT			
Structured summary	2	Provide a structured summary that includes (as applicable): background, objectives, eligibility criteria, sources of evidence, charting methods, results, and conclusions that relate to the review questions and objectives.	1
INTRODUCTION			
Rationale	3	Describe the rationale for the review in the context of what is already known. Explain why the review questions/objectives lend themselves to a scoping review approach.	1
Objectives	4	Provide an explicit statement of the questions and objectives being addressed with reference to their key elements (e.g., population or participants, concepts, and context) or other relevant key elements used to conceptualize the review questions and/or objectives.	4
METHODS			
Protocol and registration	5	Indicate whether a review protocol exists; state if and where it can be accessed (e.g., a Web address); and if available, provide registration information, including the registration number.	4
Eligibility criteria	6	Specify characteristics of the sources of evidence used as eligibility criteria (e.g., years considered, language, and publication status), and provide a rationale.	4
Information sources	7	Describe all information sources in the search (e.g., databases with dates of coverage and contact with authors to identify additional sources), as well as the date the most recent search was executed.	4
Search	8	Present the full electronic search strategy for at least 1 database, including any limits used, such that it could be repeated.	4
Selection of sources of evidence†	9	State the process for selecting sources of evidence (i.e., screening and eligibility) included in the scoping review.	5
Data charting process	10	Describe the methods of charting data from the included sources of evidence (e.g., calibrated forms or forms that have been tested by the team before their use, and whether data charting was completed independently or in duplicate) and any processes for obtaining and confirming data from investigators.	Not Applicable
Data items	11	List and define all variables for which data were sought and any assumptions and simplifications made.	Not Applicable
Critical appraisal of individual sources of evidence	12	If completed, provide a rationale for conducting a critical appraisal of included sources of evidence; describe the methods used and how this information was used in any data synthesis (if appropriate).	5
Synthesis of results	13	Describe the methods of handling and summarizing the data that were charted.	5
RESULTS			
Selection of sources of evidence	14	Give numbers of sources of evidence screened, assessed for eligibility, and included in the review, with reasons for exclusions at each stage, ideally using a flow diagram.	5
Characteristics of sources of evidence	15	For each source of evidence, present characteristics for which data were charted and provide the citations.	7
Critical appraisal within sources of evidence	16	If completed, present data on critical appraisal of included sources of evidence (see item 12).	9
Results of individual sources of evidence	17	For each included source of evidence, present the relevant data that were charted that relate to the review questions and objectives.	7
Synthesis of results	18	Summarize and/or present the charting results as they relate to the review questions and objectives.	12
DISCUSSION			
Summary of evidence	19	Summarize the main results (including an overview of concepts, themes, and types of evidence available), link to the review questions and objectives, and consider the relevance to key groups.	14
Limitations	20	Discuss the limitations of the scoping review process.	17
Conclusions	21	Provide a general interpretation of the results with respect to the review questions and objectives, as well as potential implications and/or next steps.	18
FUNDING			
Funding	22	Describe sources of funding for the included sources of evidence, as well as sources of funding for the scoping review. Describe the role of the funders of the scoping review.	18
